# Micro-Current Stimulation Suppresses Inflammatory Responses in Peptidoglycan-Treated Raw 264.7 Macrophages and *Propionibacterium acnes*-Induced Skin Inflammation via TLR2/NF-κB Signaling Pathway

**DOI:** 10.3390/ijms23052508

**Published:** 2022-02-24

**Authors:** Hana Lee, Donghyun Hwang, Minjoo Lee, Jinho Lee, Seungkwan Cho, Tack-Joong Kim, Han Sung Kim

**Affiliations:** 1 Department of Biomedical Engineering, Yonsei University, Wonju 26493, Korea; hanah4378@naver.com (H.L.); ggubuing@naver.com (D.H.); mj9732@naver.com (M.L.); 2 Division of Biological Science and Technology, Yonsei University, Wonju 26493, Korea; drlogos@naver.com (J.L.); ktj@yonsei.ac.kr (T.-J.K.); 3 CELLOGIN Inc., Wonju 26354, Korea; biecsk@naver.com

**Keywords:** acne, peptidoglycan, *Propionibacterium acnes*, micro-current stimulation, TLR2/NF-κB signaling

## Abstract

Acne is a common inflammatory disorder of the human skin and a multifactorial disease caused by the sebaceous gland and *Propionibacterium acnes* (*P. acnes*). This study aimed to evaluate the anti-inflammatory effect of micro-current stimulation (MC) on peptidoglycan (PGN)-treated raw 264.7 macrophages and *P. acnes*-induced skin inflammation. To specify the intensity with anti-inflammatory effects, nitric oxide (NO) production was compared according to various levels of MC. As the lowest NO production was shown at an intensity of 50 μA, subsequent experiments used this intensity. The changes of expression of the proteins related to TLR2/NF-κB signaling were examined by immunoblotting. Also, immunofluorescence analysis was performed for observing NF-κB p65 localization. All of the expression levels of proteins regarding TLR2/NF-κB signaling were decreased by the application of MC. Moreover, the application of MC to PGN-treated raw 264.7 cells showed a significant decrease in the amount of nuclear p65-protein. In the case of animal models with *P. acnes*-induced skin inflammation, various pro-inflammatory cytokines and mediators significantly decreased in MC-applied mice. In particular, the concentration of IL-1β in serum decreased, and the area of acne lesions, decreased from the histological analysis. We suggest for the first time that MC can be a novel treatment for acne.

## 1. Introduction

Acne is a chronic inflammatory symptom occurring in the pilosebaceous units on the face, resulting in the formation of comedones, papules, pustules, and scarring [1]. Among several known pathogenic mechanisms, it is known that *Propionibacterium acnes (P. acnes)*, comedonal bacteria, plays a crucial role in the pathogenesis of acne by inducing monocyte and/or macrophages to activate pro-inflammatory mediators [2]. For this reason, there are common treatment options including antibiotics and anti-inflammatory drugs for acne treatment [3,4,5]. However, long-term treatment of medication is to be less effective by the development of resistant strains of *P. acnes* and can induce common side effects including gastrointestinal discomfort, esophagitis, and photosensitivity [6,7,8]. Therefore, new treatments of acne have been suggested, which offer durable responses and have minimal side effects. The most representative treatment would be light and laser therapy such as blue/red light and the 1450 nm diode laser with anti-bactericidal and anti-inflammatory effects on acne as a non-drug treatment. Such treatments have been cleared by the Food and Drug Administration (FDA) [9,10,11,12,13].

Recently, there are many studies which have demonstrated that electrical stimulation has beneficial effects on the skin such as skin barrier formation, facial skin care, wound healing, and psoriasis. In particular, micro-current stimulation, generally known as electrical stimulation with low amperage (<1000 μA), has been reported to be involved in all wound healing phases divided into three-phase including inflammation, proliferation, and remodeling [14,15,16,17]. Among them, we mainly focused on anti-inflammatory and anti-bacterial effects which are most closely related to acne. Previous studies showed that the electric current in the range of 70–90 μA using galvanic zinc-copper particles reduced the edema response and excessive inflammatory response in a rat ear skin model [18]. Similarly, anti-inflammation effects have been observed for electrical stimulation with the intensity of 50 μA applied to human wounds induced by ultraviolet irradiation [19]. Taken together, all of them were effective on the inflammatory response at relatively low levels of current intensity. The anti-bacterial effects of electrical stimulation can be explained by the changes in the electrical polarity of the skin induced by bacteria. Regardless of Gram-positive bacteria or Gram-negative bacteria, they are known to carry a negative charge in the wound area [20]. For this reason, the positive polarity of the electric field decreases their motility, resulting in reducing the bacteria-induced inflammation response [21]. Based on this, we hypothesized that micro-current stimulation with low levels of current may alleviate acne, a common skin disease accompanied by inflammatory reactions.

*P. acnes*, as well as peptidoglycan (PGN) from Gram-positive bacteria, has been mainly used for the efficacy of various drugs or treatment in acne-induced inflammatory reactions [22,23,24,25,26]. *P. acnes* plays a crucial role in the inflammatory phase of acne through activating innate immunity via toll-like receptor 2 (TLR2), one member of transmembrane proteins that respond to particular pathogen-associated molecular patterns (PAMPs) such as bacterial cell wall components [27,28,29]. Since PGN is a representative molecule of PAMPs, which is a major cell-wall component of Gram-positive bacteria including *P. acnes*, it has been used to trigger TLR2-mediated immune responses in several cell lines including macrophages, sebocytes, and keratinocytes [22,23,30,31,32]. TLR2-mediated immune responses accomplish their signals through the myeloid differentiation factor 88 (MyD88)-dependent pathway, followed by activating nuclear factor-κB (NF-κB) subsequently regulating cytokine gene expression. Therefore, the investigation with downregulation of TLR2 signaling pathway activated with *P. acnes* or PGN is needed to determine if micro-current stimulation can improve the inflammatory response associated with acne.

In this study, we investigated whether micro-current stimulation can suppress TLR-2/NF-κB signaling and reduce several inflammatory cytokines in PGN treated-macrophage. Additionally, the anti-inflammatory effects of the micro-current stimulation with the specific intensity on *P. acnes*-induced skin inflammation were evaluated in the mice model.

## 2. Results

### 2.1. Micro-Current Stimulation Could Decrease the NO Production and TLR2 Related Protein Expression

**Figure 1:**
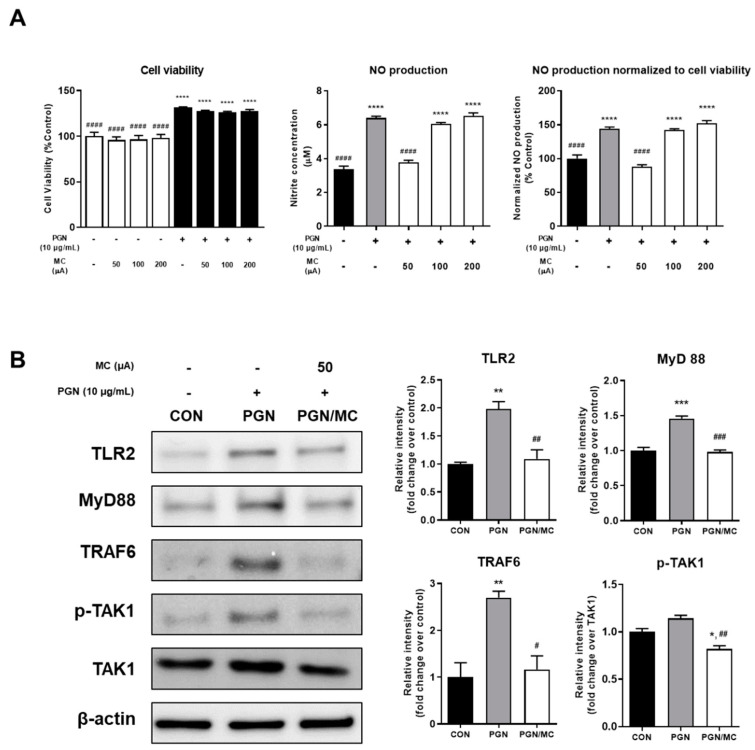
(**A**) To determine the cytotoxicity of PGN or MC in Raw 264.7 cells, the presence or absence of PGN treatment or various levels of MC were applied to the Raw 264.7 cells. The cell was subsequently cultured for 12 h and incubated further with WST-1 reagent for an additional 1 h. To compare NO production at various levels of MC, Raw 264.7 cells were pre-treated with PGN (10 μg/mL) for 30 min and applied with MC for 1 h. After 12 h, the amount of NO production was measured by the Griess assay. **** *p* < 0.0001 vs. negative control. #### *p* < 0.0001 vs. positive control. The values shown represent the mean ± SD of triplicate measurements of separate experiments. Values are shown as % of the negative control. (**B**) Immunoblot analysis of TLR2 related proteins on Raw 264.7 cells following the application of MC. The graph represents the quantitative level of the proteins. * *p* < 0.05, ** *p* < 0.01, *** *p* < 0.001 vs. control group. # *p* < 0.05, ## *p* < 0.01, ### *p* < 0.001 vs. positive control.

The cytotoxicity of PGN and micro-current stimulation (MC) on raw 264.7 cells was determined by using WST-1 assay after 12 h. As shown in Figure 1A, cell viability was not affected for all intensities of MC. However, the PGN-treated cells, a positive control, showed a cell viability of about 131%. PGN-treated cells with the application of MC did not show a significant difference from the only PGN-treated cells.

To investigate MC could inhibit the NO production stimulated by PGN in raw 264.7 cells, raw 264.7 cells were incubated at 37 °C for 30 min in a medium containing PGN (10 μg/mL). After that, MC was applied for 1 h. Since the cell viability showed a significant difference depending on the presence or absence of PGN treatment, the NO production rate was normalized in consideration of the cell viability after 12 h, and the value was expressed as % Control. NO production of PGN-treated cells was strongly increased. However, NO production stimulated by PGN was inhibited in only the 50 μA MC group (*p* < 0.0001) (Figure 1A).

Western blot analysis revealed that the protein expression levels of TLR2, MyD88, TRAF6, and p-TAK1 increased due to PGN treatment in Raw 264.7 cells. In contrast, the PGN/MC group significantly reduced the PGN-induced TLR2, MyD88, TRAF6, and p-TAK1 protein levels.

### 2.2. MC Could Decrease Activity of NF-κB and Related Protein Expression

**Figure 2:**
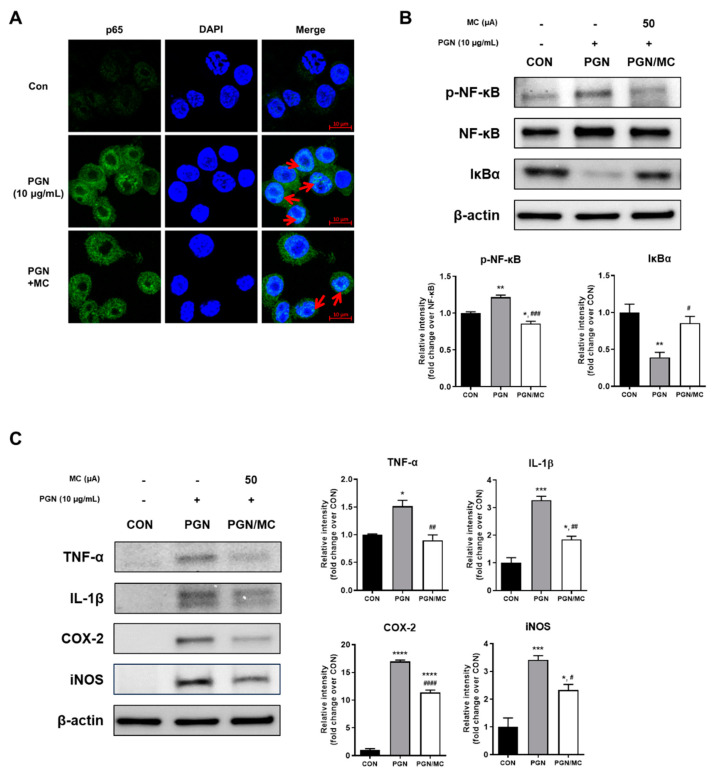
(**A**) The translocation of NF-κB (p65) to the nucleus was analyzed by confocal microscopy. MC-applied cells showed a reduced translocation of p65 to the nucleus. Red arrows indicate the region with relatively high p65 intensity. (**B**) Immunoblot analysis of NF-κB-related proteins on Raw 264.7 cells following the application of MC. Cells were treated in the presence or absence of MC for 1 h and pretreated with or without 10 μg/mL of PGN for 30 min. The cell lysate was analyzed by immunoblot using p-NF-κB, NF-κB, and IκBα antibodies. (**C**) Immunoblot analysis of pro-inflammatory cytokines or mediators on Raw 264.7 cells following the application of MC. The cell lysate was analyzed by immunoblot using TNF-α, IL-1β, COX-2, and iNOS antibodies. The graph represents the quantitative level of the proteins. * *p* < 0.05, ** *p* < 0.01, *** *p* < 0.001, **** *p* < 0.0001 vs. control group. # *p* < 0.05, ## *p* < 0.01, ### *p* < 0.001, #### *p* < 0.0001 vs. positive control.

In order to examine the decreased activity of NF-κB after application of MC, we further determined the localization of p65-protein, a major subunit of NF-κB, via immunocytochemistry even after PGN treatment. Compared to the control approach, we observed the translocation of p65 to the nucleus of Raw 264.7 cells after PGN stimulation (Figure 2A, second panel). Due to PGN treatment, p65 expression showed high activity inside of the nucleus, and it appeared to surround the edge of the nucleus rather than the center of the nucleus. Red arrows indicate the region with relatively high p65 intensity, and fewer regions in PGN + MC than in PGN (Figure 2A, third panel). Quantification of data from three independent experiments revealed that the level of nuclear p65-protein significantly increased after exposure to PGN, whereas MC significantly decreased the level.

To examine the changes in the expression levels of NF-κB and IκBα, we evaluated the expression of proteins by immunoblot analysis. The protein amount of phosphorylated NF-κB (p-NF-κB) increased after PGN treatment, but decreasing amounts exposed to PGN and MC in Raw 264.7 cells. Correspondingly, IκBα was highly degraded in PGN-treated, while exposed to PGN and MC led to increased protein levels (Figure 2B).

In order to investigate a potential correlation between nuclear translocation of NF-κB p65 and potential alterations in the expression of NF-κB and NF-κB target protein, we further investigated expression levels of TNF-α, IL-1β, COX-2, and iNOS by western blot analysis. PGN-treated RAW264.7 cells showed increased protein expression levels of TNF-α, IL-1β, COX-2, and iNOS in comparison to control, while co-treatment with MC led to strongly attenuated protein expression (Figure 2C).

### 2.3. MC Suppressed P. acnes-Mediated Skin Inflammation in Mice

**Figure 3:**
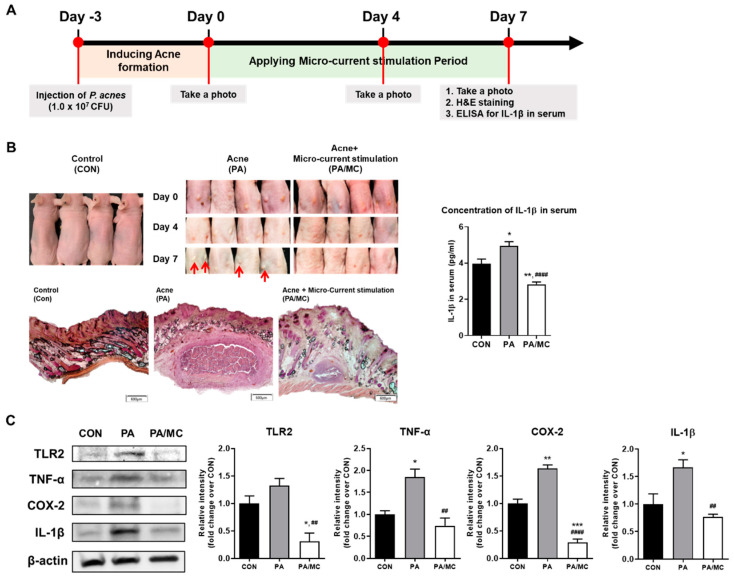
(**A**) Schematic diagram indicating the timeline for animal experiments. (**B**) Photographs of dorsal skin were taken at days zero, four, and seven in control (CON), *P. acnes*-injected mice (PA) used as a positive control, and *P. acnes*-injected mice applied MC with the intensity of 50 μA group (PA/MC). Longitudinal sections of the individual lesions for each group by H&E staining on day seven. Also, ELISA analysis was conducted to measure serum IL-1β contributing to the development of inflammatory papules, pustules, and nodules in acne. (**C**) Immunoblot analysis of TLR2 and pro-inflammatory cytokines or mediators on the tissue of acne lesions. The tissue lysate was analyzed by immunoblot using TLR2, TNF-α, COX-2, and IL-1β antibodies. * *p* < 0.05, ** *p* < 0.01, *** *p* < 0.001, vs. control group. ## *p* < 0.01, #### *p* < 0.0001 vs. positive control.

To investigate the role of micro-current stimulation in *P. acnes*-induced skin inflammation in vivo, mice were injected subcutaneously with *P. acnes* on day -three. After three days, micro-current stimulation was applied to the mice from day zero to day seven, and the mice were euthanized on day seven (Figure 3A).

*P. acnes*-injected mice began to show signs of inflammation phenotypically on day zero post-injection, such as erythema, which persisted in severity until the end of the experiment, whereas no symptoms were observed in PBS-injected mice. Interestingly, PA/MC group resulted in significantly less erythema compared to the PA group. H&E staining data showed increased dermal thickness and infiltration of inflammatory cells into the dermis in the PA group. In contrast, the PA/MC group showed significantly reduced skin thickness and decreased infiltration of inflammatory cells compared with PA group. Correspondingly, the ELISA results showed that MC significantly reduced the concentration of IL-1β level in serum (Figure 3B). Next, we evaluated the effect of MC on the expression of pro-inflammatory cytokines or mediators in the skin of *P. acnes*-injected mice. Consistent with the histological changes, immunoblot analysis also showed that the protein levels of TLR2 and inflammatory cytokines (TNF-α, COX-2, and IL-1β) were increased in the *P. acnes*-treated skin, whereas PA/MC mice have lower levels of inflammatory cytokines, compared with control (Figure 3C), suggesting that micro-current stimulation inhibited the *P. acnes*-induced skin inflammation in mice.

## 3. Discussion

There are various factors of acne such as endocrine disorder, hyper-keratinization of the follicular epithelium, abnormal follicular environment on the sebaceous glands, bacterial infection, and inflammatory response [33,34,35]. Among them, acne-related skin inflammation is mediated by an inflammatory cascade triggered by bacterial infection, especially *P. acnes*. *P. acnes*, Gram-positive anaerobic bacterium, is crucial in initiating and maintaining the inflammatory response in acne [36]. TLR-2, one of the TLRs that primarily recognizes Gram-positive bacteria, can be activated by several molecular structures such as peptidoglycans (PGNs), lipoproteins, lipoteichoic acid (LTA), and lipopolysaccharides (LPS), which are common components of the walls of various bacteria [37]. The inflammatory response to the recognition of *P. acnes* in TLR2 contributes to the inflammatory nature of acne by inducing monocytes to secrete pro-inflammatory cytokines including TNF-α, IL-1, and IL-8 [38]. Indeed, the macrophages distributed around acne lesions produce pro-inflammatory cytokines as a result of TLR-2 activation by *P. acnes* [35]. *P. acnes* also produces lipases, proteases, and hyaluronidases, all of which contribute to tissue damage [39,40,41]. For these reasons, modulating the inflammatory response of *P. acnes* has been the main treatment target for inflammatory acne.

Although the medication options are effective in this, they still represent limitations such as unwanted side effects, antibiotic resistances and recurrences [42,43]. Consequently, there is high demand to suggest a novel treatment for acne. In addition, it is necessary to develop a novel therapeutic approach which is effective and has fewer side effects, rather than relying solely on drugs. Due to such high demand, acne treatment methods using physical stimuli rather than drugs have emerged, and a representative method is using light or laser. However, studies on methods using electrical stimulation are still lacking.

Growing evidence shows that micro-current electrical stimulation exhibits anti-inflammatory effects in various diseases such as arthritis and the inflammatory phase of a chronic wound. It is known that it has various physiological effects such as inhibition of bacterial growth and healing of damaged tissues, but studies for the treatment of acne have not been confirmed [44]. It has been suggested that electrical stimulation with biphasic currents can alleviate acne through analysis of peripheral blood related to the peripheral immune system. However, there is a limit to concluding that the inflammatory response mediated by TLR2, one of the main mechanisms of acne, can be directly controlled from the results of peripheral blood analysis alone. Therefore, we used electrical stimulation of a biphasic current as one of the electrical stimulation parameters. Recently, it has been reported that pruritus can be alleviated through the TLR2/4-MyD88-NF-κB pathway using electrical stimulation in a morphine-induced pruritus mouse model [45]. However, the method used in this study is to apply electrical stimulation in an invasive manner with the intensity of 2 mA on the skin in a particular location known to control itching. Unlike this intensity, the micro-current stimulation has the advantage that the user does not feel any discomfort and non-invasive stimulation is possible since the intensity of micro-current stimulation is similar to that of the bioelectricity. So far, research on determining whether micro-current stimulation can inhibit the TLR2-mediated inflammatory response related to the main mechanism of acne has not yet been conducted. Likewise, it was necessary to study the intensity of micro-current stimulation, which has a potential anti-inflammatory effect on the acne-induced inflammatory response.

We have conducted various skin-related studies such as wound healing, hair growth promotion, and skin improvement through previous studies [17,46,47]. Consistent with other studies, it has been shown to have positive effects on the skin within the intensity range below 200 µA. Acne, a common inflammatory skin disease, was also predicted to affect even at low intensity. We evaluated which of the micro-current stimulation intensities of 50, 100, and 200 µA could have anti-inflammatory effects based on the nitric oxide (NO) production. Based on the results of observing the cell viability according to the presence or absence of PGN treatment and MC, it was found that MC did not affect the cell viability. Interestingly, it was found that PGN treatment could affect cell viability. According to the previous study in which H9c2 cardiomyocytes were treated with PGN at different concentrations and observed over time, some low-concentration-treated cells showed higher cell viability compared to negative controls, although there was no statistical significance [48]. In our study using Raw 264.7 cells, it was found that PGN treatment at a concentration of 10 µg/mL increased the cell viability at 12 h. For this reason, we normalized NO production according to the cell viability results. As shown in Figure 1A, 50 µA showed the lowest amount and rate of NO production. Accordingly, we selected the intensity with 50 µA as micro-current stimulation for all subsequent experiments.

Previous studies reported that *P. acnes* triggers activation of TLR2 signaling leading to activation of the NF-κB pathway, which is responsible for the expression of downstream genes including pro-inflammatory cytokines, chemokines, and prostaglandins. In, *P. acnes*-induced reactive oxygen species trigger activation of NF-κB and mitogen-activated protein kinase (MAPK) pathways, which are in turn induce protein expressions of inducible NO synthase (iNOS)/NO and cyclooxygenase-2/prostaglandin E2 (COX-2/PGE2) in macrophages [49]. Therefore, the inhibition of TLR2/NF-κB signaling activation has been suggested as a main therapeutic target for the anti-inflammatory strategy in *P. acnes*-induced skin inflammation [28,29,50].

In the current study, we showed that micro-current suppressed innate immune responses via TLR2/NF-κB signaling in PGN-treated macrophages. It is reported that TLR2 initiates signaling by recognizing pathogen-associated molecular patterns derived from *P. acnes* such as PGN and lipoproteins through its extracellular domain [51,52]. We have also confirmed that the expression level of TLR2 was significantly increased by about 2 times in PGN-treated cells. Subsequently, the invading signals are transported from dimerization of the extracellular domain to that of the intracellular Toll/interleukin-1 receptor domain, which recruits adaptor proteins including MyD88-adapter-like protein and MyD88 [53,54,55,56]. MyD88 causes phosphorylation of IRAK and subsequently promotes TRAF6 activation that facilitates activation of canonical NF-κB pathway through recruiting transforming growth factor-activated kinase 1 (TAK1) and TAK1-binding protein 2 (TAB2) [55,57]. Therefore, we investigated the changes in the expression level of the proteins, such as TLR2 and –related proteins (MyD88, TRAF6, and p-TAK1), involved in inducing canonical NF-κB pathway activation. As shown in Figure 1A, PGN showed a higher expression level about 1.5 to 2.5 times that of CON for them in consistent with aforementioned previous studies. However, when micro-current stimulation was applied, the expression levels of all of these proteins were significantly reduced in the PGN/MC, showing similar levels to those of CON. These results could be implied that micro-current stimulation may downregulate subsequent signaling, canonical NF-κB pathway activation.

NF-κB is one of the important transcription factors, which is responsible for numerous inflammatory genes related to acne and the innate immune response [35,54,55,56,58,59]. NF-κB pathway is mainly carried out by the interaction between NF-κB and IκB in the cytosol. In detail, NF-κB is dissociated from IκB by serine-specific IκB kinase (IKK) complex leading to phosphorylation and degradation of IκB, then it is translocated to the nucleus to perform the function of a transcription factor [56,60,61]. To determine the effect of micro-current stimulation on this signaling, our present study investigated the expression level of NF-κB-related proteins by western blotting and the location of NF-κB p65 by immunofluorescence staining. As shown in Figure 2B, the protein expression of p-NF-κB significantly increased, whereas that of IκBα significantly decreased in the PGN group. On the contrary, the application of MC not only reduced the protein expression of p-NF-κB but also enhanced the protein expression of IκBα. Consistent with these results, the same trend could be identified in the result of immunofluorescence staining as shown in Figure 2A. We found that NF-κB p65-positive staining was mainly localized in the cytoplasm and shifted to the nuclear with PGN. It seems that p65 activity is increased in the nucleus due to PGN, and the activated region is located at the inner edge of the nucleus rather than the center of the nucleus. These characteristics were similar to the immunofluorescence results of previous studies in which macrophages were treated with TLR3 ligand and PGN [62]. These findings suggest that the pattern is different from the p65 translocation expression pattern of LPS, which appeared to be activated by spreading relatively uniformly within the nucleus [63,64,65]. Interestingly, the application of MC reduced the PGN-induced translocation of NF-κB p65. These findings implied that MC can modulate the inflammatory signaling cascade through the regulation of NF-κB activation.

In the interaction between TLR2 and *P. acnes*, NF-κB acts as an essential TLR2 downstream signal that has a major impact on inflammatory responses in acne by releasing various pro-inflammatory cytokines such as COX-2, iNOS, IL-1β, and TNF-α [65]. These cytokines play a major role in the follicular hyper-keratinization and inflammatory responses of acne [66]. As shown in Figure 2C, our data showed that PGN increased the expression of these cytokines at the protein level in Raw 264.7 cells. Interestingly, it was shown that the expression of these pro-inflammatory cytokines was significantly suppressed when MC was applied. Taken together, these data implied that MC could bring a protective effect on PGN-induced skin inflammation through suppression of TLR2/NF-κB axis in vitro.

**Figure 4:**
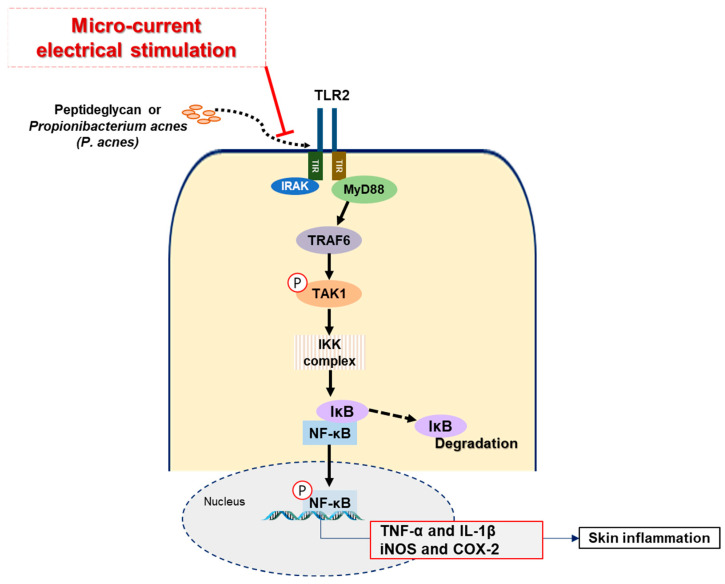
A model of MC in the suppression of PGN or *P. acnes*-induced inflammatory responses. MC exerts anti-inflammatory effects in acne lesions by downregulating TLR2/NF-κB signaling pathways.

In order to determine the therapeutic effects of MC in vivo, we used *P. acnes*-injected mouse model, a well-known animal model of acne [67]. Three days after intradermal injection of bacteria into the dorsal skin, we could observe typical inflammatory symptoms caused by *P. acnes*, such as cutaneous erythema and thickening of the skin. For this reason, the MC was implemented three days after injection and observed for seven days (Figure 3A). It is known that several inflammatory cytokines are responsible for the exacerbation of inflammatory symptoms after acne occurs, so we mainly observed the expression of cytokine protein and histological changes in acne lesions according to MC. In the results of H&E staining, MC-applied lesions showed markedly reduced swelling, the number of infiltrated inflammatory cells, and granulomatous response compared with lesions injected with only *P. acnes*. Several inflammatory cytokines such as TNF-α, IL-1β, COX-2 are known to be responsible for follicular hyper-keratinization and are involved in the process of changing these acne lesions [66]. TNF-α is one of the factors expressed in the early stages of the inflammatory and immune reaction in acne and causes inflammatory responses such as vasodilatation, edema, and fever [68,69,70,71]. IL-1β is contributed to the development of inflammatory papules, pustules, and nodules in acne. The production of IL-1β by *P. acnes* is mediated by activation of the NACHT, LRR, and PYD domains-containing Protein 3(NLRP3)-inflammasome triggers strong inflammatory responses [72]. For these reasons, many studies have noted IL-1β as a novel potential therapeutic target in acne [66,72,73]. COX-2, one of the inflammatory mediators, plays a crucial role in the production of prostaglandins and lipid mediators contributing to the development of inflammatory responses. Increased production of COX-2 leads to sebaceous gland hyperplasia and enhanced sebum production. It means that COX-2 plays an important role in sebocyte biology regarding acne. In this study, MC significantly inhibited the expression levels of TNF-α, IL-1β, and COX-2, in accordance with the in vitro findings. Among them, IL-1β is known to show high content in serum in people with acne. Our results of measuring IL-1β level in serum (Figure 3B) show a lower level in MC-applied animals compared with animals injected with only *P. acnes*. The expression level of TLR-2, involved in the secretion of these cytokines and mediators, was also significantly inhibited according to MC. Based on our results and previous findings, we proposed a possible mechanism of micro-current stimulation in PGN- or *P. acnes*-mediated inflammation (Figure 4).

## 4. Materials and Methods

### 4.1. Materials

RAW 264.7 cells (ATCC^®^ TIB-71^TM^) and *Cutibacterium acnes* (ATCC^®^ 6919™) were purchased from American Type Culture Collection, ATCC (Manassas, VA, USA). Low-glucose Dulbecco’s modified Eagle medium (DMEM) was purchased from Welgene (Daegu, South Korea). Penicillin-streptomycin (PS), fetal bovine serum (FBS), and peptidoglycan (PGN) were purchased from Sigma-Aldrich Chemical Co. (St. Louis, MO, USA). EZ-Cytox, cell viability assay kit, was purchased from the Daeil Labservice (Seoul, Korea). Lysis buffer was purchased from iNtRON Biotechnology Inc. (Seongnam, Korea). Protease and Phosphatase Inhibitor Mini Tablets and BCA assay kit were purchased from Thermo Scientific (Rockford, IL, USA). IL-1β Mouse ELISA Kit (BMS6002) was purchased from Invitrogen (Carlsbad, CA, USA).

### 4.2. Micro-Current Electrical Stimulation (MC)

In the in-vitro experiments, we equalized all of MC with biphasic square pulse and a frequency of 10 Hz to compare the effects of different intensities of MC. To compare nitric oxide production according to the different intensities of MC, we applied with the MC of 50, 100, and 200 µA levels per the whole culture well for 1 h. In other in vitro experiments, we only applied with the MC of 50 µA that was relatively effective from the result of nitric oxide production.

Subsequently, an animal experiment was conducted using a 50 µA level with the custom-made electrodes made of conductive metal and hydrogel. Two electrodes were respectively attached to the upper and lower parts of the lesion, applying 40 min of MC each day for 7 days.

### 4.3. Cell Culture and Preparation of Bacteria

Raw 264.7 cells were maintained in low glucose DMEM supplemented with 10% FBS and 1% PS at 37 °C in a humidified incubator under 5% CO_2_.

*P. acnes* (ATCC 6919) was purchased from American Type Culture Collection (ATCC, Manassas, VA) and grown in Modified Reinforced Clostridial medium (BD, MD, USA) at 37 °C under anaerobic condition (5% H_2_, 5% CO_2_, and 90% N_2_) until reaching OD 600 = 1.0 (logarithmic growth phase). *P. acnes* were harvested by centrifugation at 4000 rpm for 15 min at 4 °C to remove supernatant. Bacterial pellets were washed three times and resuspended to a concentration of 10^7^ CFU/20 μL in PBS for the experiments.

### 4.4. Cell Viability

Cell viability of raw 264.7 cells was determined using the EZ-cytox cell viability assay kit. Groups were divided according to the presence or absence of PGN treatment and MC application, and EZ-cytox assay solution was added to the medium after 12 h. After 1 h incubation, cell viability was measured at a wavelength of 450 nm on a microplate reader (Epoch, BioTek Instruments, Winooski, VT, USA).

### 4.5. Determination of Nitric Oxide (NO) Production

Raw 264.7 cells (5 × 10^5^ cells per well) were seeded on a 6-well plate for 24 h and pre-treated with peptidoglycan (PGN, 10 μg/mL) for 30 min and subsequently applied with the MC of 50, 100, and 200 µA level for 1 h under the serum-free medium. After 12 h additional incubation, the quantity of nitrite accumulated in the culture medium was measured via the Griess reaction (Weissman and Gross, 2001) [74]. Briefly, 100 µL of supernatant from each well was mixed with 100 µL of Griess reagent (1% sulfanilamide and 0.1% naphthyl ethylenediamine dihydrochloride in 5% phosphoric acid) in a separate 96-well plate. After an incubation of 10 min at RT, the absorbance at 540 nm was measured in a microplate reader (Epoch, BioTek Instruments, Winooski, VT, USA). The measured NO production was normalized for each cell viability result, and the normalized value was expressed as % of negative control.

### 4.6. Animals

All experimental protocols (YWCI-201910-016-01) were approved by the Institutional Animal Care and Use Committee, Yonsei University, and were conducted under the Guide for the National Institutes of Health guidelines. Five-week-old BALB/c-nu mice (30 mice) were randomly divided into three groups (ten mice/group), including Control group (CON group; Intradermal injection into the back skin with 20 μL PBS), *P. acnes* group (PA group; Intradermal injection with 1 × 10^7^ CFU of *P. acnes* per 20 μL PBS) and Acne with micro-current stimulation group (PA/MC group; Intradermal injection with 1 × 10^7^ CFU of *P. acnes* per 20 μL PBS and applying with MC).

After the bacteria injection, the acne induction period was given for three days. After the acne induction period, the PA/MC group was applied with the MC of 50 µA level for seven days. At the end of the experimental period, these animals were euthanized by cervical dislocation after collecting blood samples through cardiac puncture under anesthesia. And their back skins were used for histological analysis and immunoblotting analysis.

### 4.7. Immunoblotting and Immunofluorescence Analysis

Cells and skin tissues were lysed using lysis buffer (iNtRon Biotech, Seoul, South Korea) for 1 h. Total cell and tissue lysates were centrifuged at 12,000× *g* at 4 °C for 10 min to obtain supernatants. After bicinchoninic acid (BCA, Sigma) protein quantification assay, equal amounts of protein were separated by SDS-PAGE gels and then transferred onto the PVDF membrane, blocked using 3% Blotting Grade Blocker in Tris-buffered Saline-Tween 20 (TBS-T) for 1 h, and then incubated overnight with the specific antibodies at 4 °C with a 1:1000 dilution. In this experiment, primary antibodies were used including: TLR2 (#13744, Cell Signaling Technology), MyD88 (#4283, Cell Signaling Technology), TRAF6 (#ab33915, Abcam), p-TAK1 (#9339, Cell Signaling Technology), TAK1 (#4505, Cell Signaling Technology), p-NF-κB (#3033, Cell Signaling Technology), NF-κB (#8242, Cell Signaling Technology), IκBα (#4812, Cell Signaling Technology), TNF-α (#11948, Cell Signaling Technology), IL-1β (#31202, Cell Signaling Technology), COX-2 (#12282, Cell Signaling Technology), iNOS (#13120, Cell Signaling Technology), and β-actin (#4967, Cell Signaling Technology).

After washing three times with TBS-T, these membranes were incubated with a 1:5000 dilution of anti-Rabbit IgG, HRP-linked Secondary Antibody (#7074, Cell Signaling Technology) for 1 h at RT followed by three times of washes with TBS-T. The protein-antibody complexes were visualized with Amersham™ ECL™ Select Western Blotting Detection Reagent (RPN2235, GE Healthcare). Images were recorded with an Image Quant LAS 500 (GE healthcare, UK). Band densities were quantified using Image J software (1.52a version, National Institutes of Health, Bethesda, MD, USA).

For the immunofluorescence analysis, cells were grown on 6-well plate and coating cover slides and fixed with 3.7% paraformaldehyde for 15 min in PBS. The membrane was permeabilized by treating cells for 1 min with 0.1% Triton X-100 in PBS. The cells were then placed in blocking solution (3% BSA in PBS) for 30 min at RT. Cells were incubated with a 1:1000 dilution of anti-p65 primary antibody (#8242, Cell Signaling Technology) for overnight at 4 °C. After washing twice, they were incubated with a 1:400 dilution of the Alexa Flour 488 (excitation/emission = 495/519 nm, green, Invitrogen, CA, USA) for 1 h at RT. After washing three times, cells were counterstained with DAPI and cover-glass were mounted using VECTASHIELD Antifade Mounting Medium with DAPI(LS-J1033, LSBio, Inc., Seattle, WA, USA). Immunolabeling was examined using a LSM700 confocal microscope (Carl-Zeiss, Oberkochen, Germany). To observe subcellular localization of p65, images were merged using the ZEN Lite software (Zeiss, Oberkochen, Germany). In the merged image of each cell, the intensity profile was investigated only within the DAPI region, and the region with relatively high p65 intensity was indicated by a red arrow.

### 4.8. Enzyme-Linked Immunosorbent Assay (ELISA)

The concentration of IL-1β in serum was measured with IL-1β Mouse ELISA Kit (BMS6002, Invitrogen, Carlsbad, CA, USA). ELISA was performed according to the manufacturer’s instructions.

### 4.9. Histological Analysis

Dorsal skin lesions were excised and immediately fixed with 10% formalin and dehydrated in 10%, 15%, and 20% sucrose solution. Fixed skins were embedded in a cryomold with optimal cutting temperature (OCT) compound (FSC 22 Clear, Leica Biosystems, Wetzlar, Germany) and sectioned using a Leica CM1860 cryostat (Leica Microsystem Ltd., Wetzlar, Germany) at −20 °C. The sections were stained with hematoxylin and eosin (H&E) and then visualized by a light microscope (Olympus DP80, Olympus Optical Co., Ltd., Tokyo, Japan).

### 4.10. Statistical Analysis

All results were presented as the means ± SD. The statistical software package SPSS 25 (IBM SPSS Statistics, SPSS Inc., Chicago, IL, USA) was used to evaluate the effects of MC. The statistical analysis was determined by one-way analysis of variance (ANOVA) followed by Tukey’s test. Differences were considered to be significant for values of *p* < 0.05.

## 5. Conclusions

In conclusion, we suggest that micro-current stimulation with the intensity of 50 μA may contribute to alleviating *P. acnes*- and PGN-induced inflammatory response. Thereby, it might be a useful method for anti-inflammatory treatment associated with acne. To our knowledge, we suggest for the first time that micro-current electrical stimulation has a potential as a treatment for the alleviating PGN- or *P. acnes*-induced inflammatory responses.

## Data Availability

Not applicable.
